# Improved glucose recovery from durian peel by alkaline-catalyzed steam pretreatment

**DOI:** 10.7717/peerj.12026

**Published:** 2021-08-18

**Authors:** Abraham Kusi Obeng, Duangporn Premjet, Siripong Premjet

**Affiliations:** 1 Department of Biotechnology, Faculty of Biosciences, University for Development Studies, Tamale, Northern Region, Ghana; 2 Center of Excellence in Research for Agricultural Biotechnology, Department of Agricultural Science, Faculty of Agriculture, Natural Resources and Environment, Naresuan University, Muang Phitsanulok, Phitsanulok, Thailand; 3 Department of Biology, Faculty of Science, Naresuan University, Muang Phitsanulok, Phitsanulok, Thailand

**Keywords:** Autoclave, Agricultural waste, Durian peel, Enzyme hydrolysis, Glucose recovery, Sodium hydroxide

## Abstract

Durian (*Durio zibethinus* Murr.) peel, as agricultural waste, is a potential under-utilized lignocellulosic biomass that is sufficiently available in Thailand. In this study, durian peel from monthong (*D*. *zibethinus* Murr. cv. Monthong) and chanee (*D*.*zibethinus* Murr. cv. Chanee) were subjected to pretreatment with sodium hydroxide (NaOH) under autoclaving conditions to improve glucose recovery. The effect of NaOH concentration (1%, 2%, 3%, and 4%) and autoclave temperature (110 °C, 120 °C, and 130 °C) was investigated based on the amount of glucose recovered. The optimal NaOH concentration and autoclave temperature were determined to be 2% and 110 °C, respectively, under which maximum glucose (36% and 35% in monthong and chanee peels, respectively) was recovered. Glucose recovery was improved by about 6-fold at the optimal pretreatment condition for both pretreated monthong and chanee when compared to the untreated durian peels. Scanning electron microscopy (SEM) showed great changes to the surface morphology of pretreated durian peel from the two cultivars. X-ray diffraction (XRD) analysis also revealed a rise in cellulose crystallinity index (CrIs) after pretreatment. A combination of mild NaOH concentration and autoclaving is a very effective pretreatment technique for maximum glucose recovery from durian peel.

## Introduction

**Figure 1:**
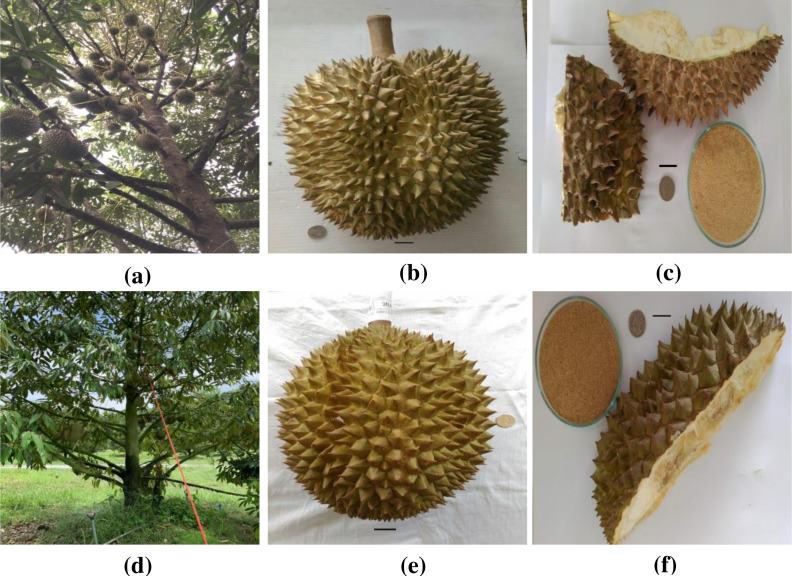
Photograph of (A) monthong tree (B) monthong fruit (C) monthong peel (D) chanee tree (E) chanee fruit (F) chanee peel. Bar = 2 cm. Source: Siripong Premjet (2020).

Durian (*Durio zibethinus* Murr.) is a very popular economic fruit grown in Southeast Asia. It is a very important fruit in Thailand, yielding around 635, 031 tons in 2017 (Siwina & Leesing, 2021). Among the various durian cultivars in Thailand, only a few, including MonThong (*D*. *zibethinus* Murr. cv. MonThong) and Chanee (*D*. *zibethinus* Murr. cv. Chanee) (refer to Fig. 1), are grown commercially (Pinsorn et al., 2018). The fruit is large in size with ovoid, obovoid, or oblong shape, strong aroma, green to brownish pericarp color, and thorn-covered peel (refer to Fig. 1) (Aziz & Mhd Jalil, 2019). The fruit pulp, which is the edible portion, is fleshy, thick, juicy, and tasty. The pulp is highly nutritious and can be used as supplement for nutritional and health purposes. It is rich in vitamins, minerals, fats, dietary fiber, and essential amino acids (Wanwimolruk et al., 2015). The fleshy pulp represents 20–35% of the fruit weight while 65% is the peel (refer to Fig. 1). Fresh consumption of the fruit and minimal processing, therefore, produce a large amount of raw agricultural waste.

Durian peel is a lignocellulosic biomass resource that can be converted to bioethanol in Thailand. The conversion of lignocellulosic biomass to biofuel has attracted global attention especially in tropical countries like Thailand (Siwina & Leesing, 2021). Using durian peel to produce bioethanol could provide an alternative use of this raw agricultural waste. However, durian peel, as lignocellulosic biomass, has low biodegradability because of its complex fibrous structure. The cellulose component is highly crystalline and embedded in hemicellulose and lignin, severely restricting enzymatic and microbial accessibility, thereby resulting in low sugar production (Kumar et al., 2020). Thus, to enhance enzymatic hydrolysis and increase glucose production, an efficient pretreatment process to open up the complex fibrous structure of the durian peel is very crucial. An important criterion in determining the optimum pretreatment condition(s) is the amount of glucose recovered after enzymatic hydrolysis. The optimum pretreatment condition(s) for enhancing the efficiency of enzymatic hydrolysis may be severe enough to reduce glucose recovery and subsequently lead to low bioethanol yield. Maximum glucose should therefore be recovered at the optimum pretreatment condition(s) for fermentation into bioethanol (Yoo et al., 2017).

Pretreatment is the most critical step during the process of cellulosic ethanol production. Various pretreatment methods classified into biological, physical, chemical, and physicochemical have been developed to improve biodegradability of lignocellulosic biomass (Wang et al., 2020). Of the reported pretreatment methods, chemical pretreatment with alkaline reagents is considered a viable technique for industrial applications (Haldar & Purkait, 2020). Compared to acid pretreatment, alkaline pretreatment is eco-friendly, non-corrosive, and produces less inhibitory products. Alkali-based pretreatment techniques are currently being extensively used in bioconversion processes because they are very effective in removing acetyl groups in xylan, degrading lignin, decreasing cellulose crystallinity, and increasing the porosity of lignocellulosic biomass (Chong et al., 2017). Among the various alkali reagents, sodium hydroxide (NaOH) is reported to be the most selective for lignin removal. Pretreatment with NaOH has received particular attention as a technology closest to commercialization (Sarbishei, Goshadrou & Hatamipour, 2020). However, the effectiveness of NaOH pretreatment can be further improved by the addition of an appropriate heating method.

Autoclave heating technology is advantageous because the heating process is homogeneous in the chamber and the temperature is stable throughout the process. It is carried out under pressure in a closed chamber devoid of air (Scherzinger & Kaltschmitt, 2019). Steam is introduced into the autoclave chamber under pressure, forcing out air and rapidly increasing the temperature in the chamber to compliment the effect of NaOH (Ahmad Khan, Booker, & Yu, 2015). Autoclave-assisted alkaline pretreatment is considered very efficient in destroying the recalcitrant structure of lignocellulosic biomass and improving access to cellulose. It has been successfully used for the pretreatment of wheat bran and oat hulls (Debiagi et al., 2020), bamboo shoot shell (Chong et al., 2017), switchgrass (Xu et al., 2010), and corn straw (Huang et al., 2017). In all these studies, a general increase in the glucose content of the biomass was reported after autoclave-assisted alkaline pretreatment. However, the effect of the studied pretreatment conditions on glucose recovery was not analyzed in these researches. To take full advantage of the effect of autoclave-assisted alkaline pretreatment, crucial process conditions such as alkaline concentration and autoclave temperature must be optimized and their effect on glucose recovery analyzed.

In this study, the effect of NaOH along with autoclaving on glucose production from durian peel is reported. The influence of NaOH concentration and autoclave temperature is investigated. Morphological changes are also studied using a scanning electron microscope (SEM) and X-ray diffraction (XRD).

## Materials & Methods

### Schematic overview of experimental program

**Figure 2:**
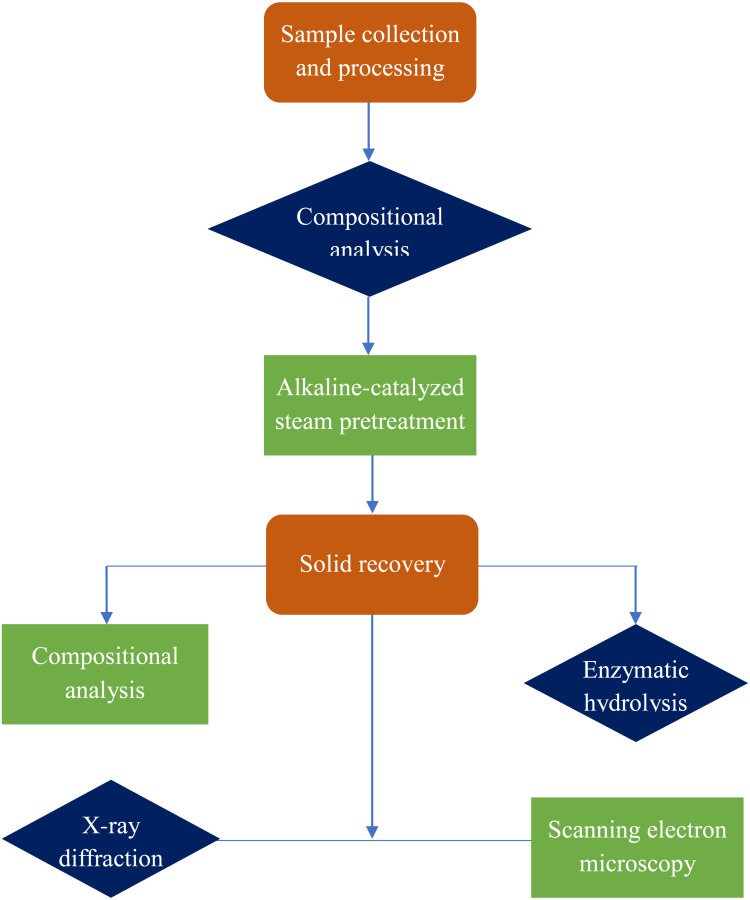
Schematic overview of glucose recovery from durian peel.

A general flow diagram of the alkaline-catalyzed steam pretreatment process to recover glucose from durian peel is depicted in Fig. 2. The process mainly includes sample collection and processing, pretreatment, compositional analysis of raw and pretreated durian peel, and enzymatic hydrolysis. Morphological changes after pretreatment were also studied using X-ray diffraction and scanning electron microscopy.

### Collection and processing of durian peel

Collection and processing of durian peel were carried out as previously described in Obeng, Premjet & Premjet (2019). Monthong and chanee peels (refer to Fig. 1) were sampled from Phitsanulok Province, Thailand. The durian peel was cut into smaller fragments, open dried for 10 days, and powdered using a wood miller (SM 100; Rtsch, Rheinis-che StraBe 36-D-42781, Haan, North Rhine-Westphalia, Germany). The powder was subjected to sieving through a mesh with a pore size of 150–300 µm and subsequently put into airtight plastic bags at room temperature prior to further study.

### Alkaline-catalyzed steam pretreatment of durian peel

A pretreatment experiment was conducted by varying only one factor at a time while keeping the other fixed. Firstly, the effect of NaOH concentration (1%, 2%, 3%, and 4% w/v) was investigated at an autoclave (TOMY SX-500; Tomy Digital Biology Co., Ltd., Tagara, Nerima-ku, Tokyo, Japan) temperature of 120 °C and 1.0 g/8.0 mL for solid/liquid ratio. Further, durian peel was pretreated by autoclaving at 110 °C, 120 °C, and 130 °C with the best NaOH concentration and 1.0 g/8.0 mL for solid/liquid ratio. The pretreatment process was carried out in a 125 mL Erlenmeyer flask for 60 min. The contents of each flask were thoroughly mixed and tightly covered with aluminum foil before autoclaving. After pretreatment, the pretreated material was quickly placed on ice to cool and subsequently filtered. The solid phase was thoroughly washed with distilled water till a pH of 7.0 was attained and used for further analysis.

### Compositional analysis of durian peel

The structural carbohydrates and lignin (Sluiter et al., 2012) contents of durian peel were analyzed according to the National Renewable Energy Laboratory (NREL, Golden, CO, USA) analytical procedures. The contents of monosaccharides were determined as described in Obeng, Premjet & Premjet (2019) using the high-performance liquid chromatography (HPLC) system (Agilent 1100, Agilent Technologies, Waldbronn, Germany). The HPLC system consisted of a G1362A (Agilent Technologies, Waldbronn, Germany) refractive index detector (RID) and Bio-Rad Aminex HPX-87P column (Bio-Rad Laboratories, Inc., Hercules, CA, USA). The HPLC system was run at a temperature of 80 °C and the injection of 20 µL of each sample. Filtered HPLC-grade water was used as an eluent at a flow rate of 0.6 mL/min.

### Enzymatic hydrolysis

Enzymatic hydrolysis was carried out following the method described in Obeng, Premjet & Premjet (2018). The untreated and pretreated durian peel was hydrolyzed by suspending 0.1 g of peel (dry weight) in 10 mL of digestion solution. The solution was made up of 0.05 M sodium citrate buffer (pH 4.8, w/v), 2% (w/v) sodium azide, and an enzyme cocktail of 30 filter paper units (FPU) of cellulase (celluclast 1.5 L, Sigma-Aldrich, St. Louis, MO, USA) together with 60 U β-glucosidase (Oriental Yeast Co., Ltd., Tokyo, Japan) per gram of dry biomass. The reaction was conducted in a 50 mL Erlenmeyer flask at a temperature of 50 °C and fixed rotation of 150 rpm (revolution per minute) with a rotary shaker (Innova 4340; New Brunswick Scientific Company, Edison, NJ, USA) for 72 h. Hydrolysates were collected periodically (12 h, 24 h, 48 h, and 72 h) and assayed for glucose concentration using the HPLC. The enzymatic hydrolysis efficiency and glucose recovery were calculated as follows:

\begin{eqnarray*}\mathrm{HE}\hspace*{2.22198pt}(\text{%})= \frac{\text{Glucose released}\hspace*{2.22198pt}(\mathrm{g})}{1.11\times \text{Glucan in initial biomass}\hspace*{2.22198pt}(\mathrm{g})} \times 100 \end{eqnarray*}

\begin{eqnarray*}\mathrm{GR}\hspace*{2.22198pt} \left( \text{%} \right) =[\mathrm{SR}\hspace*{2.22198pt} \left( \text{%} \right) \times \text{Glucan}\hspace*{2.22198pt} \left( \text{%} \right) \times 1.11\times \mathrm{HE}\hspace*{2.22198pt} \left( \text{%} \right) ]\times 100 \end{eqnarray*}

where HE is hydrolysis efficiency, GR is glucose recovery, and SR is solid recovery. The glucan to glucose conversion factor is 1.11.

### Scanning electron microscopy (SEM)

Scanning electron microscopy (SEM) analysis was performed with an LEO 1455VP microscope (Zeiss, Gottingen, Germany). The untreated and pretreated durian peel was freeze-dried and mounted on aluminum stubs using double-sided tape for visualization. The surface of the biomass was then sputter-coated with a thin gold layer and visualize under the microscope at a magnification of 500 × and beam accelerating voltage of 20 kV.

### X-ray diffraction (XRD)

The untreated and pretreated durian peel were washed with acetone three times, dried at 32 °C, and powdered to the size of 150 µm. The crystallinity of the biomass was then investigated with PANalytical X’pert Pro, PW 3040/60 diffractometer (Almelo, The Netherlands) by scanning from 2 *θ* = 10° to 40° at the rate of 0.02° s^−1^. The crystallinity index (CrI) was calculated as follows:

\begin{eqnarray*}\mathrm{CrI}\hspace*{2.22198pt}(\text{%})= \frac{{\mathrm{I}}_{002}-{\mathrm{I}}_{\mathrm{am}}}{{\mathrm{I}}_{002}} \times 100 \end{eqnarray*}

where I_002_ is the peak intensity of the crystalline peak and I_am_ is the peak for the amorphous cellulose.

### Statistical analysis

All experiments in this study were conducted on a dry weight basis and included a minimum of three replicates. Data reported are the mean values with their standard deviations. The data were statistically analyzed using one-way analysis of variance (ANOVA) at a 5% significance level. Tukey’s test was used for multiply comparisons. Microsoft Excel 2010 was used for drawing graphs.

## Results

### Compositional analysis

Compositional analysis (dry weight) of the untreated durian peel in the current study revealed the presence of mainly glucan, xylan, and lignin (refer to Tables 1 and 2). These components represent approximately 70% and 69% of the total content of monthong and chanee peels, respectively.

**Table 1:** Composition of durian peel pretreated with various NaOH concentrations at 120 °C.

**Cultivar (dw)**	NaOH Conc. % (w/v)	**Glucan** % (w/w)	**Xylan** % (w/w)	**Lignin** % (w/w)	Delignification % (w/w)	**CrIs** (%)
Monthong	Untreated	43.43 ± 0.37^e^	12.05 ± 0.15^a^	14.86 ± 0.17^a^	–	28.01
	1	56.29 ± 0.17^d^	10.39 ± 0.12^b^	8.80 ± 0.12^b^	62.29 ± 0.46^d^	38.43
	2	59.12 ± 0.57^c^	8.28 ± 0.09^c^	4.78 ± 0.12^c^	80.83 ± 0.26^c^	40.61
	3	62.28 ± 0.66^b^	7.77 ± 0.11^d^	4.35 ± 0.13^d^	85.70 ± 0.30^b^	41.33
	4	64.43 ± 0.29^a^	7.15 ± 0.06^e^	3.93 ± 0.09^e^	88.75 ± 0.37^a^	44.62
Chanee	Untreated	41.63 ± 0.45^e^	11.92 ± 0.19^a^	15.39 ± 0.21^a^	–	20.72
	1	53.53 ± 0.28^d^	10.48 ± 0.17^b^	9.33 ± 0.16^b^	60.28 ± 0.38^d^	25.63
	2	56.46 ± 0.35^c^	9.05 ± 0.09^c^	5.67 ± 0.15^c^	77.80 ± 0.26^c^	28.53
	3	60.24 ± 0.27^b^	8.42 ± 0.13^d^	4.92 ± 0.13^d^	84.12 ± 0.21^b^	30.20
	4	62.31 ± 0.33^a^	7.36 ± 0.09^e^	4.33 ± 0.10^e^	87.87 ± 0.17^a^	37.71

**Notes.**

Data are expressed as means ± standard deviation (*n* = 3). Means in the same column with different superscript letters (^a,b,c,d,e^) differ statistically at *p* < 0.05; dw represents dry weight.

**Table 2:** Composition of durian peel pretreated with 2% NaOH at various temperatures.

**Cultivar (dw)**	Temp. (°C)	**Glucan** % (w/w)	**Xylan** % (w/w)	**Lignin** % (w/w)	Delignification % (w/w)	**CrIs** (%)
Monthong	Untreated	43.43 ± 0.37^d^	12.05 ± 0.15^a^	14.86 ± 0.17^a^	–	28.01
	110	55.27 ± 0.24^c^	9.71 ± 0.13^b^	5.07 ± 0.08^b^	76.91 ± 0.35^c^	35.40
	120	59.12 ± 0.57^b^	8.28 ± 0.09^c^	4.78 ± 0.12^bc^	80.83 ± 0.26^b^	40.61
	130	62.11 ± 0.89^a^	6.35 ± 0.22^d^	4.53 ± 0.16^c^	85.85 ± 0.33^a^	43.03
Chanee	Untreated	41.63 ± 0.45^d^	11.92 ± 0.19^a^	15.39 ± 0.21^a^	–	20.72
	110	54.31 ± 0.22^c^	9.65 ± 0.08^b^	5.87 ± 0.12^b^	74.11 ± 0.32^c^	27.22
	120	56.46 ± 0.35^b^	9.05 ± 0.09^c^	5.67 ± 0.15^b^	77.80 ± 0.26^b^	28.53
	130	60.30 ± 0.39^a^	6.02 ± 0.10^d^	5.17 ± 0.14^c^	85.16 ± 0.41^a^	35.63

**Notes.**

Data are expressed as means ± standard deviation (*n* = 3). Means in the same column with different superscript letters (^a,b,c,d^) differ statistically at *p* < 0.05; dw represents dry weight.

### Impact of sodium hydroxide concentration

Pretreatment by different concentrations of NaOH had a significant effect on the chemical composition of both monthong and chanee peels (refer to Table 1). The glucan content of both cultivars significantly (*p* = 0.0001) increased with a concomitant decrease (*p* = 0.0001) in both xylan and lignin as NaOH concentration increased at the set temperature of 120 °C. When compared to untreated durian peel, a maximum of 1.5-fold increase in glucan content was observed after pretreatment with 4% (w/v) NaOH for both cultivars. A decrease in lignin content was more obvious in peels from both cultivars. Delignification in the range of approximately 62% to 89% for pretreated monthong peel and 60% to 88% for pretreated chanee peel were observed.

**Figure 3:**
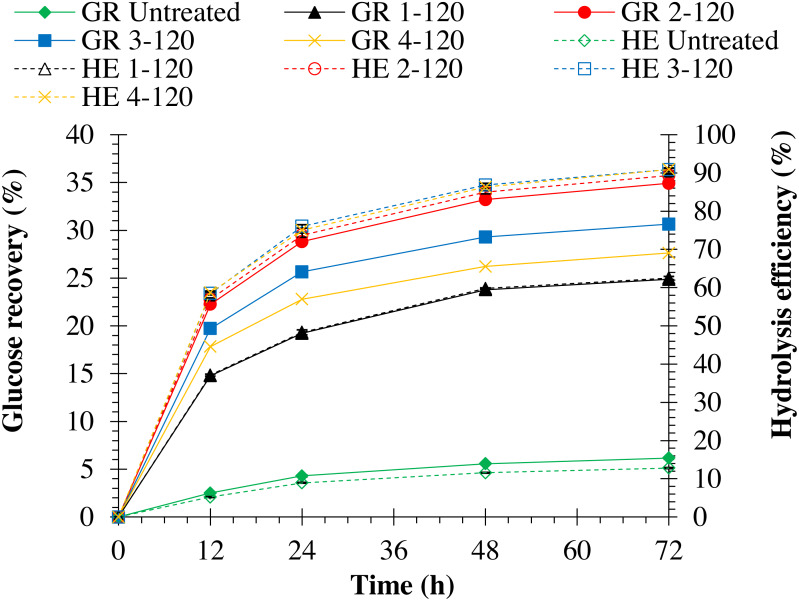
Enzymatic hydrolysis of untreated and pretreated monthong peel at different NaOH concentrations. Note: GR Untreated represents glucose recovery for raw peels, GR 1-120 represents glucose recovery for peels pretreated with 1% NaOH at 120 °C, GR 2-120 represents glucose recovery for peels pretreated with 2% NaOH at 120 °C, GR 3-120 represents glucose recovery for peels pretreated with 3% NaOH at 120 °C, GR 4-120 represents glucose recovery for peels pretreated with 4% NaOH at 120 °C, HE Untreated represents hydrolysis efficiency for raw peels, HE 1-120 represents hydrolysis efficiency for peels pretreated with 1% NaOH at 120 °C, HE 2-120 represents hydrolysis efficiency for peels pretreated with 2% NaOH at 120 °C, HE 3-120 represents hydrolysis efficiency for peels pretreated with 3% NaOH at 120 °C, and HE 4-120 represents hydrolysis efficiency for peels pretreated with 4% NaOH at 120 °C.

**Figure 4:**
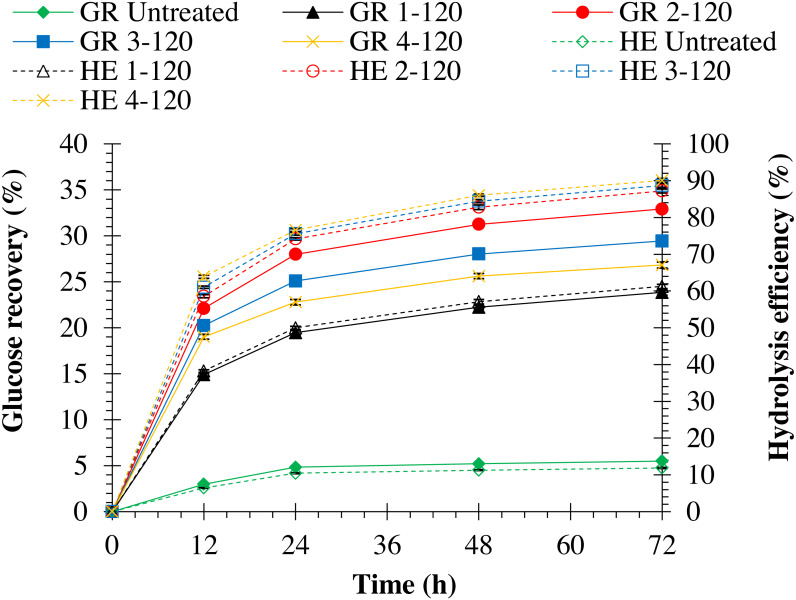
Enzymatic hydrolysis of untreated and pretreated chanee peel at different NaOH concentrations. Note: GR Untreated represents glucose recovery for raw peels, GR 1-120 represents glucose recovery for peels pretreated with 1% NaOH at 120 °C, GR 2-120 represents glucose recovery for peels pretreated with 2% NaOH at 120 °C, GR 3-120 represents glucose recovery for peels pretreated with 3% NaOH at 120 °C, GR 4-120 represents glucose recovery for peels pretreated with 4% NaOH at 120 ° C, HE Untreated represents hydrolysis efficiency for raw peels, HE 1-120 represents hydrolysis efficiency for peels pretreated with 1% NaOH at 120 °C, HE 2-120 represents hydrolysis efficiency for peels pretreated with 2% NaOH at 120 °C, HE 3-120 represents hydrolysis efficiency for peels pretreated with 3% NaOH at 120 °C, and HE 4-120 represents hydrolysis efficiency for peels pretreated with 4% NaOH at 120 °C.

Hydrolysis efficiency of pretreated monthong and chanee was significantly (*p* = 0.0001) improved when NaOH concentration was increased (refer to Figs. 3 and 4). Maximum hydrolysis efficiency (90.89%) was observed by pretreating monthong peel with 4% (w/v) NaOH concentration, which was similar (*p* = 1.000) to that (90.85%) observed by pretreatment with 3% (w/v) NaOH concentration (refer to Fig. 3). However, maximum (*p* = 0.027) hydrolysis efficiency (90.09%) was observed by pretreating chanee peel with 4% (w/v) NaOH concentration (refer to Fig. 4). Glucose recovery in pretreated peels from both cultivars was significantly (*p* = 0.0001) improved following enzymatic hydrolysis. Maximum glucose from pretreated monthong (34.92%) and chanee (32.92%) were significantly (*p* = 0.0001) recovered when peels were exposed to 2% (w/v) NaOH concentration. However, reduction in the glucose recovered from both cultivars was observed when pretreatment of peels was conducted at higher (3% and 4% w/v) NaOH concentrations. Considering substantial recovery of glucose and maximum delignification, 2% NaOH (w/v) is observed as an optimum dosage with 60 min of reaction time for pretreatment of durian peel from both cultivars.

### Impact of autoclave temperature

Autoclave heating in this study contributed to durian peel with significantly (*p* = 0.0001) lower lignin, xylan, and higher glucan contents than those of their respective untreated peel biomass (refer to Table 2). Increasing autoclave temperature resulted in decreasing lignin and xylan contents in monthong and chanee peels. In contrast, the relative amount of glucan in durian peel increased when autoclave temperature was increased. Durian peel subjected to 130 °C presented the lowest lignin content of approximately 86% and 85% delignification for monthong and chanee peels, respectively.

**Figure 5:**
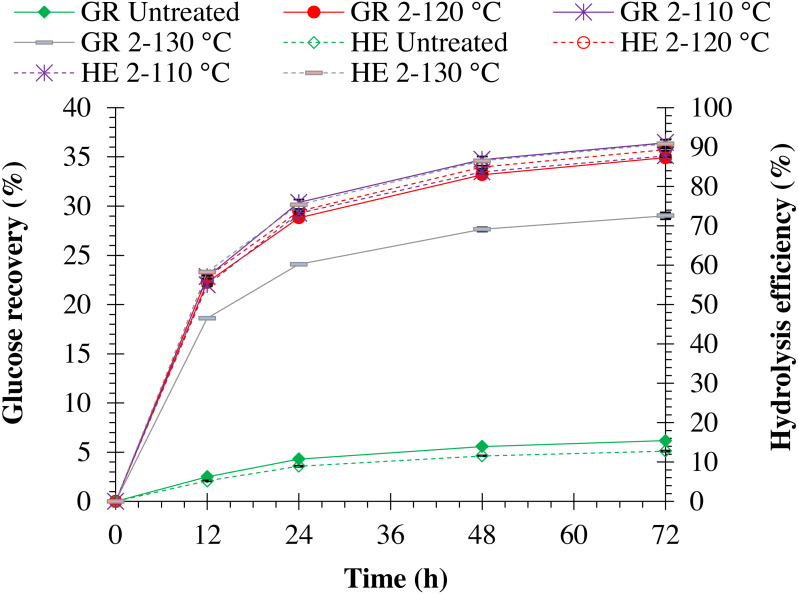
Enzymatic hydrolysis of untreated and pretreated monthong peel at different autoclave temperatures. Note: GR Untreated represents glucose recovery for raw peels, GR 2-120 represents glucose recovery for peels pretreated with 2% NaOH at 120 °C, GR 2-110 represents glucose recovery for peels pretreated with 2% NaOH at 110 °C, GR 2-130 represents glucose recovery for peels pretreated with 2% NaOH at 130 °C, HE Untreated represents hydrolysis efficiency for raw peels, HE 2-120 represents hydrolysis efficiency for peels pretreated with 2% NaOH at 120 °C, HE 2-110 represents hydrolysis efficiency for peels pretreated with 2% NaOH at 110 °C, and HE 2-130 represents hydrolysis efficiency for peels pretreated with 2% NaOH at 130 °C.

**Figure 6:**
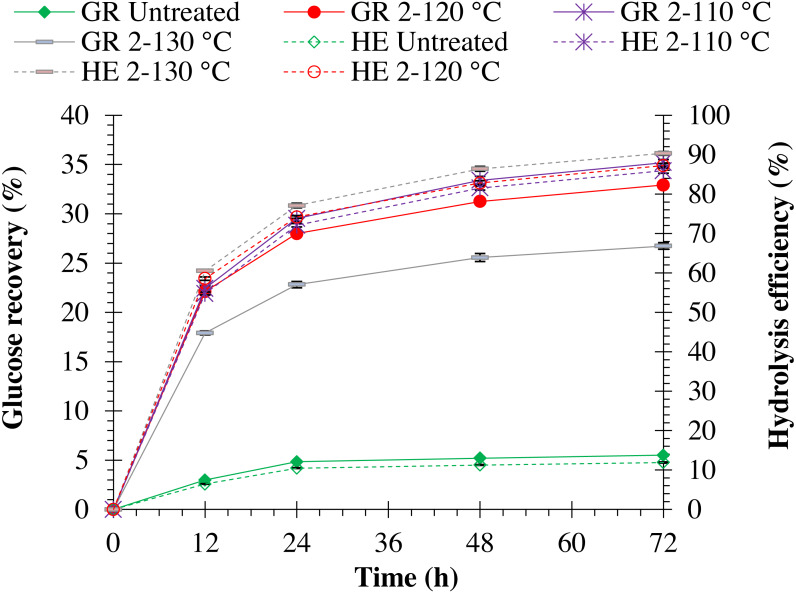
Enzymatic hydrolysis of untreated and pretreated chanee peel at different autoclave temperatures. Note: GR Untreated represents glucose recovery for raw peels, GR 2-120 represents glucose recovery for peels pretreated with 2% NaOH at 120 °C, GR 2-110 represents glucose recovery for peels pretreated with 2% NaOH at 110 °C, GR 2-130 represents glucose recovery for peels pretreated with 2% NaOH at 130 °C, HE Untreated represents hydrolysis efficiency for raw peels, HE 2-120 represents hydrolysis efficiency for peels pretreated with 2% NaOH at 120 °C, HE 2-110 represents hydrolysis efficiency for peels pretreated with 2% NaOH at 110 °C, and HE 2-130 represents hydrolysis efficiency for peels pretreated with 2% NaOH at 130 °C.

Increasing autoclave temperature resulted in a significant (*p* = 0.0001) increase in enzymatic hydrolysis efficiency in both pretreated monthong (refer to Fig. 5) and chanee (refer to Fig. 6) peels. The highest enzymatic hydrolysis efficiency of approximately 91% and 90% were achieved in monthong and chanee peels pretreated at an autoclave temperature of 130 °C, respectively. In contrast, increasing autoclave temperature resulted in a significant decrease (*p* = 0.0001) in glucose recovery of both pretreated monthong and chanee peels. Maximum glucose of about 36% and 35% in monthong (refer to Fig. 5) and chanee (refer to Fig. 6) peels pretreated at 110 °C, respectively, were recovered.

### Ultrastructural morphology

**Figure 7:**
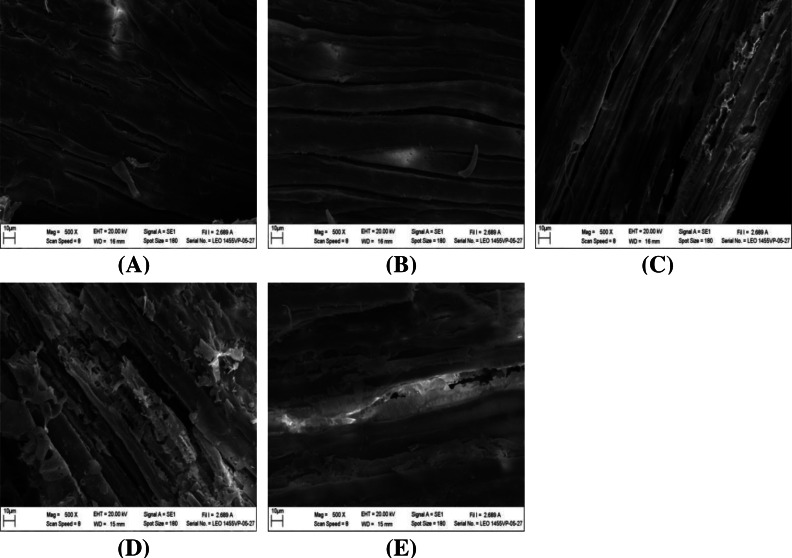
SEM images of the (A) untreated and pretreated monthong peel at (B) 1%, (C) 2%, (D) 3%, and (E) 4% NaOH concentrations.

**Figure 8:**
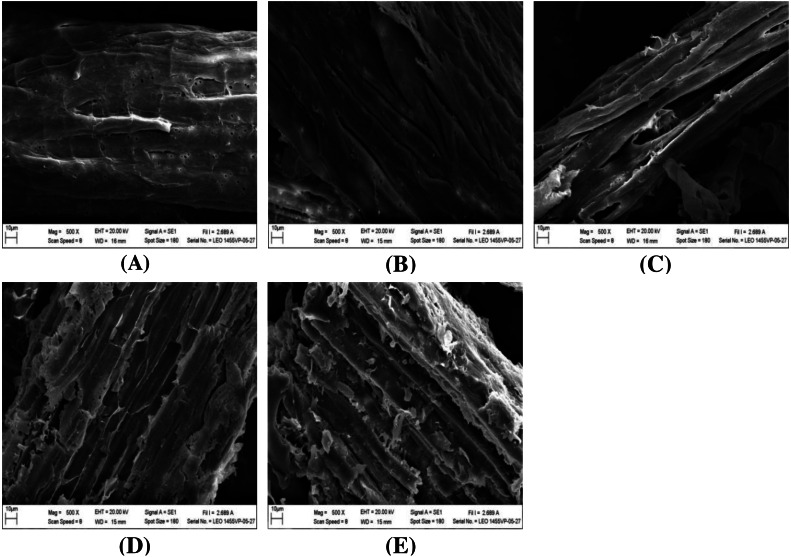
SEM images of the (A) untreated and pretreated chanee peel at (B) 1%, (C) 2%, (D) 3%, and (E) 4% NaOH concentrations.

**Figure 9:**
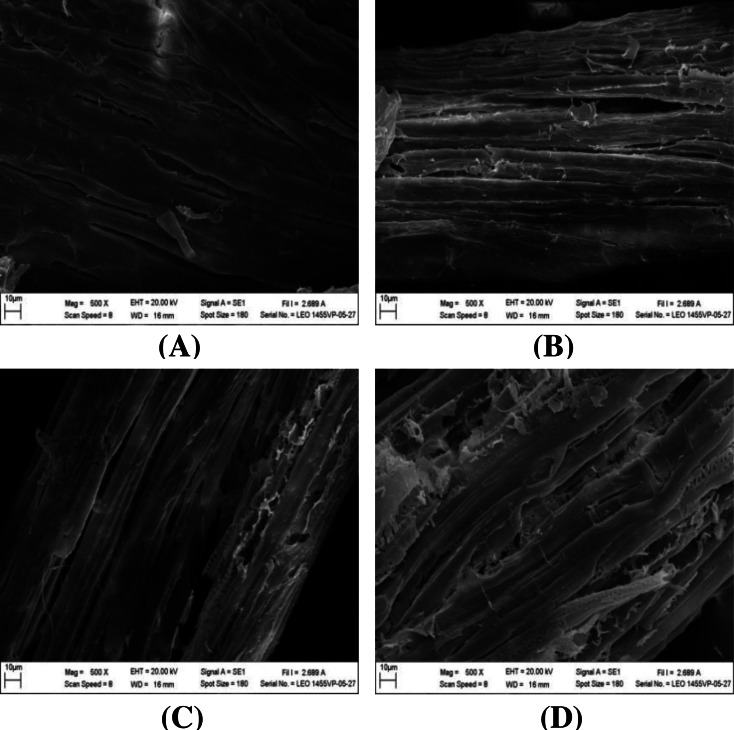
SEM images of the (A) untreated and pretreated monthong at (B) 110 °C, (C) 120 °C and (D) 130 °C.

**Figure 10:**
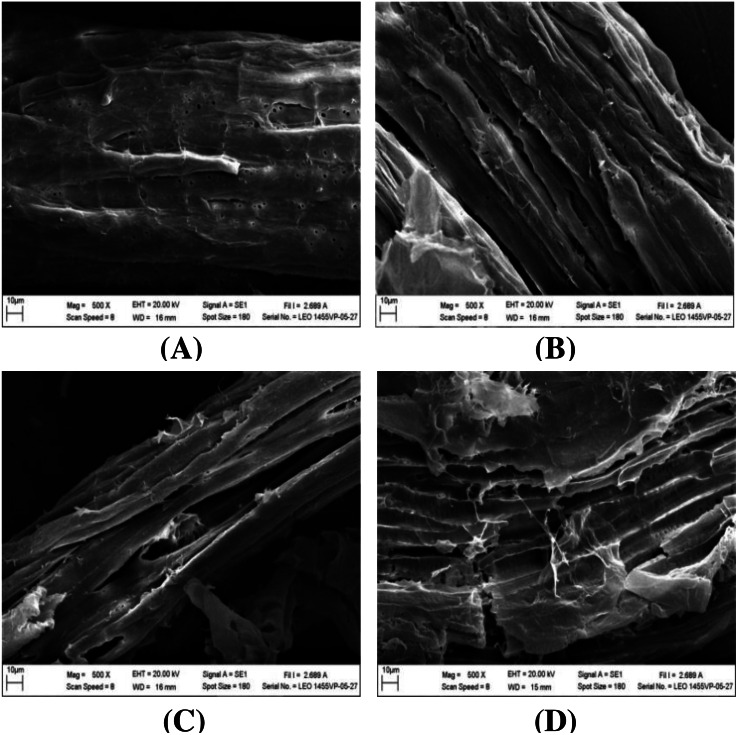
SEM images of the (A) untreated and pretreated chanee at (B) 110 °C, (C) 120 °C and (D) 130 °C.

The impact of alkaline-catalyzed steam pretreatment on structural changes in monthong and chanee peel was analyzed. The ultrastructural morphology of the untreated peel from monthong and chanee revealed structural uniformity with less surface destruction (refer to Figs. 7–10). However, pretreated monthong and chanee peels showed more destructive surface morphology (refer to Figs. 7B–7E, 8B–8E, 9B–9D, and 10B–10D). Generally, after pretreatment by different NaOH concentrations, the fibers of both monthong and chanee peels were disconnected. This resulted in the creation of long cracks on the surface of the peel biomass. The cracks became more extensive and deep as NaOH concentration was increased.

 The surface of monthong and chanee peels pretreated at the various autoclave temperatures was also rougher and more disordered. There was a fragmentation of the surface structure of peel biomass by increasing autoclave temperature. At 130 °C, the complete collapse of the peel biomass surface structure was observed.

**Figure 11:**
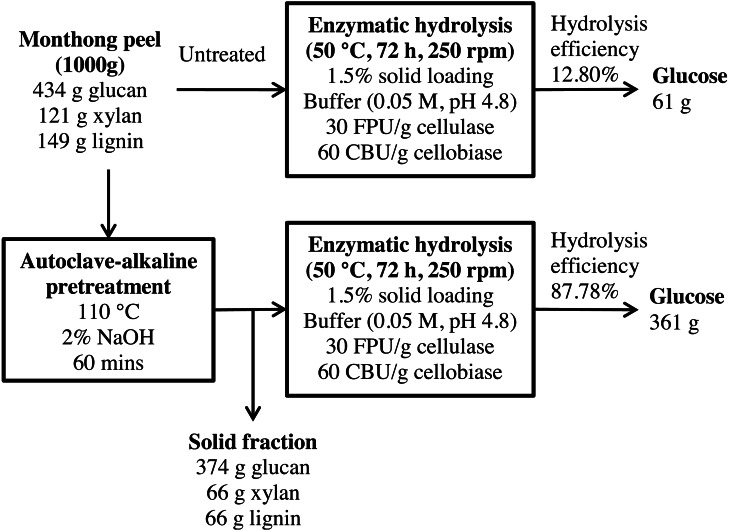
Schematic diagram of glucose recovery from monthong peel by autoclave-assisted NaOH pretreatment.

**Figure 12:**
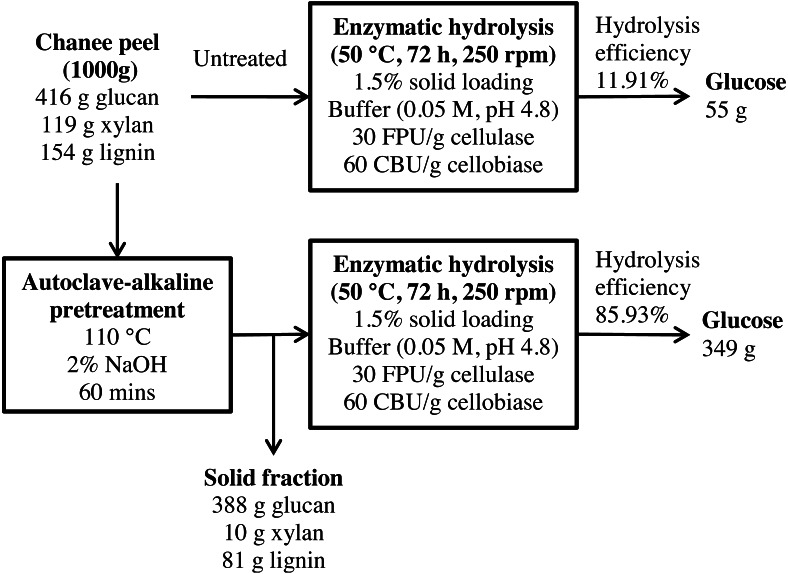
Schematic diagram of glucose recovery from chanee peel by autoclave-assisted NaOH pretreatment.

### Crystalline structure

Modifications to cellulose crystallinity (refer to Figs. S1–S4) during alkaline-catalyzed steam pretreatment of durian peel were also investigated. The crystallinity index (CrI) for both cultivars (refer to Tables 1 and 2) increased after the pretreatment process. Increasing NaOH concentration during pretreatment led to an increase in CrI for monthong and chanee (refer to Table 1). The CrI of untreated monthong peel increased from 28.01% to 44.62% after pretreatment with 4% NaOH concentration. For untreated chanee peel, CrI increased from 20.72% to 37.71% following pretreatment with a 4% NaOH. Similarly, CrI for monthong and chanee increased as autoclave temperature was increased. High CrI values of 43.03% and 35.63% for monthong and chanee, respectively, were attained at the highest autoclave temperature of 130 °C (refer to Table 2).

## Discussion

Cellulose, hemicellulose, and lignin are the main components of lignocellulosic biomass. These three main compositions, which were observed in the two cultivars of durian peel under-study, form a complex three-dimensional structure (Kumar et al., 2020). This results in low biodegradability of durian peel which can be overcome through an effective pretreatment technique (Chong et al., 2017). The presence of minor structural and non-structural components in monthong and chanee peels including galactan, arabinan, ash, and extractive has been reported in an earlier study (Obeng, Premjet & Premjet, 2018). Obvious changes in the three main compositions were observed after alkaline-catalyzed steam pretreatment in the current study (refer to Tables 1 and 2).

The significant increase in glucan content coupled with a decrease in both xylan and lignin contents in durian peel, as NaOH concentration was increased has been reported by several researchers (Coimbra et al., 2016; Li et al., 2016; Lv et al., 2017). A decrease in lignin content of durian peel from both cultivars due to various NaOH concentrations was higher than that reported by other researchers. Coimbra et al. (2016) observed 22% lignin removal after extrusion of wheat straw with 10% NaOH at 70 °C. Nieves et al. (2016) reported approximately 20% delignification following pretreatment of mango stem bark with 3% NaOH at 120 °C for 15 min. Wilkinson, Smart & Cook (2014) also reported about 32% lignin removal in 25% NaOH-pretreated brewers spent grains at 50 °C for 12 h. Solubilization of lignin and dissolution of xylan contributed to the increase in glucan content in the current study, which is in accordance with several types of researches (Lv et al., 2017; Wu et al., 2017; Yoo et al., 2017). These observations indicate the effectiveness of NaOH in the removal of lignin and dissolution of xylan during pretreatment of durian peel. Alkaline pretreatment has high delignification efficiency and is normally used in lignocellulosic biomass with high lignin content (Rezania et al., 2020). It destroys lignin structure and disrupts the ester, aryl-ether, and C–C bonds linking lignin and carbohydrate polymers to improve the biodegradability of cellulose (Kumar et al., 2020). Sodium hydroxide pretreatment has been effectively used to enhance the biodegradability of cellulose in various lignocellulosic biomass including *Pennisetum purpureum*, wheat straw, corn straw, sugarcane bagasse, Cypress, and *Pterocarpus soyauxii* (Haldar & Purkait, 2020; Wang et al., 2020).

As fractions of lignin and xylan are solubilized as a result of pretreatment with various NaOH concentrations, enzymatic hydrolysis is significantly enhanced due to exposure of cellulose to cellulase enzymes (Wu et al., 2017). Although maximum hydrolysis efficiency in both cultivars was achieved by pretreatment with 4% NaOH, less glucose was recovered. This may be due to carbohydrate solubilization. As reported earlier, a higher concentration of NaOH was favorable in achieving maximum lignin removal and hydrolysis efficiency. However, solubilization of carbohydrates may occur at high NaOH concentrations, which might cause less glucose yield (Kataria & Ghosh, 2014). This will impact negatively on the yield of ethanol during fermentation. The amount of glucose recovered is very significant in evaluating the efficacy of a pretreatment method (Premjet et al., 2018). Although the optimal pretreatment condition(s) may enhance enzymatic hydrolysis, it may also lead to less glucose recovery due to its severity (Yoo et al., 2017). Thus 2% NaOH concentration is sufficient for maximum glucose production in this study.

High autoclave temperature is more effective in disrupting the recalcitrant structure of durian peel biomass, resulting in high component removal (Debiagi et al., 2020). Previous studies have shown that this heating process in combination with a chemical pretreatment, can transform the structure and composition of lignocellulosic biomass to improve reactivity (Chong et al., 2017; Huang et al., 2017). Autoclave heating during the pretreatment process contributed significantly to enhancing the enzymatic production of glucose. Pretreated durian peel biomass at various autoclave temperatures was well hydrolyzed by cellulase enzyme. Autoclaving at 130 °C in the current study resulted in maximum hydrolysis efficiency in pretreated monthong and chanee peels. This clearly indicates that high autoclave temperature is more effective in modifying the structural and chemical properties of durian peel and improves enzymatic digestibility (Smichi et al., 2014). However, glucose recovery was significantly reduced in both cultivars at high autoclave temperatures, implying that adopting high autoclave temperatures in the current study could greatly affect glucose production from durian peel. The economics of cellulosic ethanol production is mainly dependent on the overall sugar yield (Foston & Ragauskas, 2010). Among other factors, an ideal pretreatment condition(s) should maximally recover available fermentable sugars in lignocellulosic biomass (Li et al., 2014). The ideal condition for pretreatment of durian peel in this study was identified as 2% NaOH and the temperature of 110 °C.

Changes to the ultrastructural morphology observed after pretreatment may be attributed to the collective effect of NaOH and autoclave heating. Deeper cracks were observed at high NaOH concentration and autoclave temperature, a fact that confirms the results obtained from enzymatic hydrolysis. This may be due to partial removal of the lignin-hemicellulose complex. Degradation of ester bonds and cleavage of glycosidic bonds in lignocellulosic biomass by alkali chemicals alter lignin structure, reduce the lignin-polysaccharide complex and increase the surface area and porosity (Wu et al., 2017).

The crystalline structure of cellulose is a very important feature affecting enzymatic hydrolysis efficiency. A change in crystallinity is an important parameter used in assessing the effectiveness of a particular pretreatment technique (Yan et al., 2015). High CrI observed after pretreatment might be due to removal of lignin and solubilization of xylan reported earlier, resulting in swelling of cellulose (Cai et al., 2016). This phenomenon has been generally observed and reported by several researchers (Hideno et al., 2013; Li et al., 2016; Ling et al., 2017). With the increase in NaOH concentration and autoclave temperature, CrIs of pretreated durian peel increased, affirming the removal of amorphous components and increase in cellulose content reported earlier.

A mass balance analysis of glucose recovery from durian peel was made to assess the effectiveness of the optimal pretreatment condition. The mass balance analysis was based on the glucose recovery at the optimal pretreatment conditions (2% NaOH at 110 °C for 60 min). Using 1,000 g of durian peel biomass from each cultivar, about 61 g and 55 g of glucose can be produced from monthong (refer to Fig. 11) and chanee (refer to Fig. 12) peels, respectively, after enzymatic hydrolysis at the efficiency of approximately 13% and 12%, respectively. Following pretreatment at the optimal condition, about 361 g and 349 g of glucose, representing a 6-fold increase, can be produced from monthong (refer to Fig. 11) and chanee (refer to Fig. 12) peels, respectively, after enzymatic hydrolysis at the improved efficiency of approximately 88% and 86%, respectively. Based on the outcome of the study, maximum glucose can be extracted from durian peel and fermented to bioethanol.

## Conclusions

Autoclave-assisted alkaline pretreatment is considered very effective in disrupting the lignin-carbohydrate complex of lignocellulosic biomass and improving access to cellulose. However, several researches have focused on enhancing the enzymatic hydrolysis efficiency without analyzing the effect on glucose recovery, which is a key determinant of an effective pretreatment method. The current study has clearly revealed that following pretreatment of monthong and chanee peels with NaOH under autoclaving conditions, the hydrolysis efficiency and glucose recovery of peels from both cultivars were significantly improved. Glucose recovery was enhanced by approximately 6-fold when durian peel was pretreated with mild NaOH concentration (2%) under autoclaving conditions (110 °C) for 60 min. Sodium hydroxide pretreatment under autoclaving conditions is a very efficient technique for maximum glucose recovery from durian peel. The trend of future research should be directed at fermenting the recovered glucose to bioethanol since that could not be addressed in this study.

## Supplemental Information

10.7717/peerj.12026/supp-1 Supplemental Information 1 The effect of NaOH concentrations and temperature for pretreatment and hydrolysis for glucose recovery of chanee durian peels

10.7717/peerj.12026/supp-2 Supplemental Information 2 The effect of NaOH concentrations and temperature for pretreatment and hydrolysis for glucose recovery of monthong durian peels

10.7717/peerj.12026/supp-3 Supplemental Information 3 X-ray diffraction (XRD) spectra of the cellulose-rich portions of the untreated and petreated monthong at various NaOH concentrations

10.7717/peerj.12026/supp-4 Supplemental Information 4 X-ray diffraction (XRD) spectra of the cellulose-rich portions of the untreated and petreated chanee at various NaOH concentrations

10.7717/peerj.12026/supp-5 Supplemental Information 5 X-ray diffraction (XRD) spectra of the cellulose-rich portions of the untreated and petreated monthong at various autoclave temperatures

10.7717/peerj.12026/supp-6 Supplemental Information 6 X-ray diffraction (XRD) spectra of the cellulose-rich portions of the untreated and petreated chanee at various autoclave temperatures

## Additional Information and Declarations

### Data Availability

The following information was supplied regarding data availability: Raw data is available in the Supplemental Files.
