# Supplementary figures and images for: Improved glucose recovery from durian peel by alkaline-catalyzed steam pretreatment
Source: PeerJ. 2021 Aug 18;9:e12026. doi: 10.7717/peerj.12026 (PMC8380032; doi:10.7717/peerj.12026)

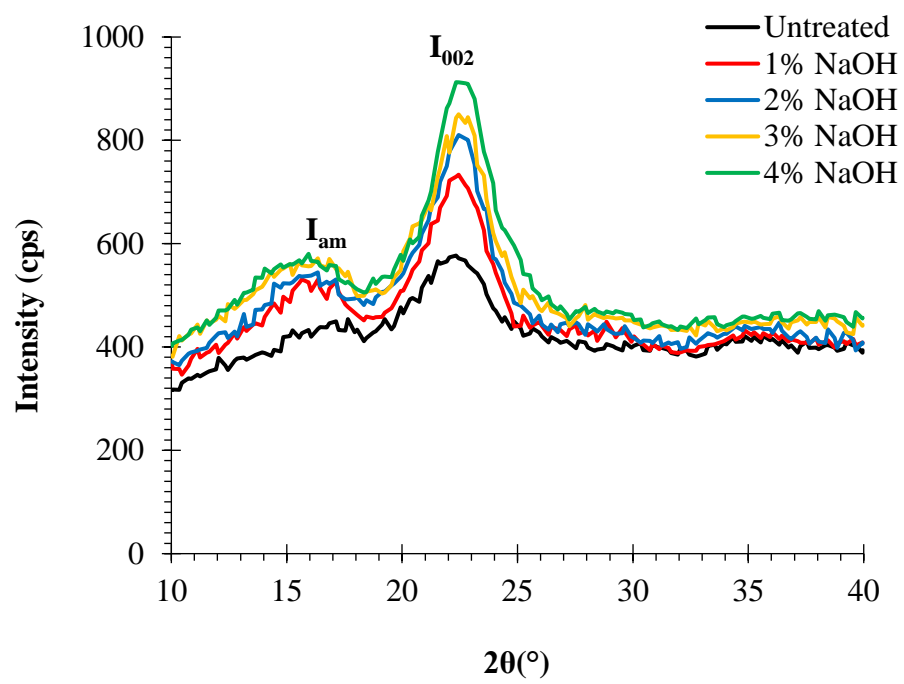

Supplement: Supplemental Information 3 [file peerj-09-12026-s003.pdf]

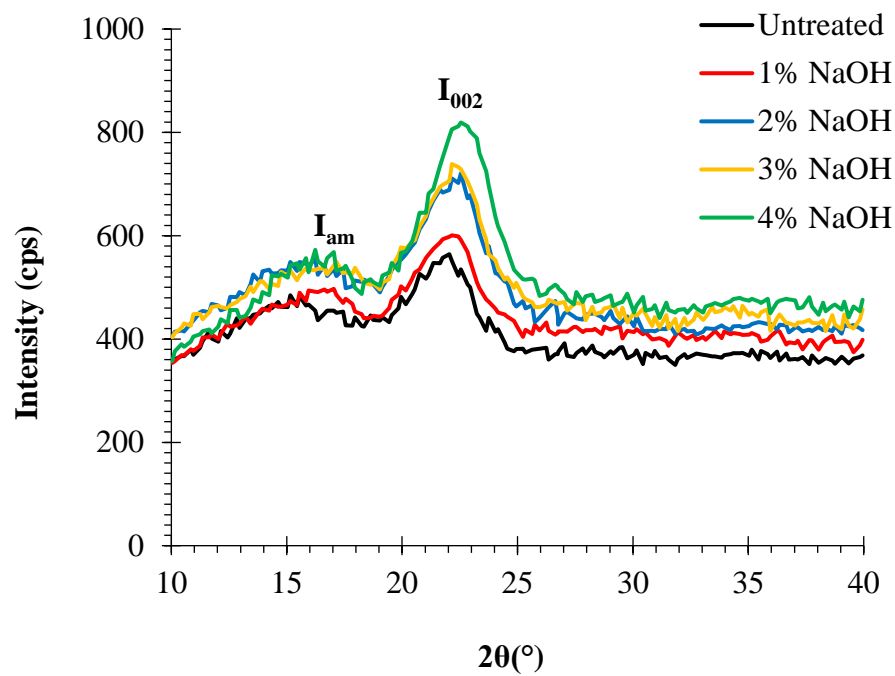

Supplement: Supplemental Information 4 [file peerj-09-12026-s004.pdf]

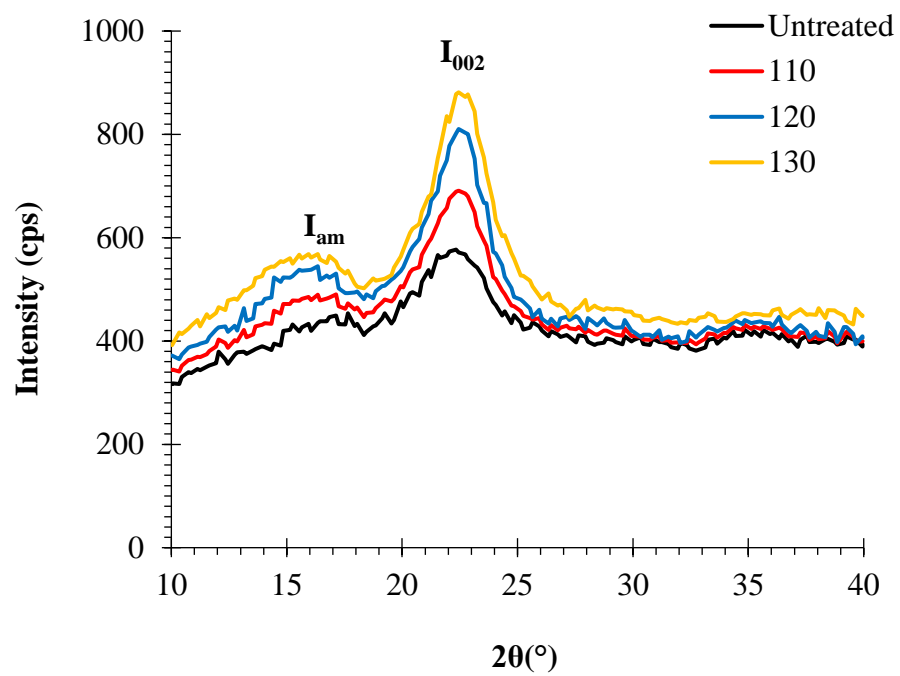

Supplement: Supplemental Information 5 [file peerj-09-12026-s005.pdf]

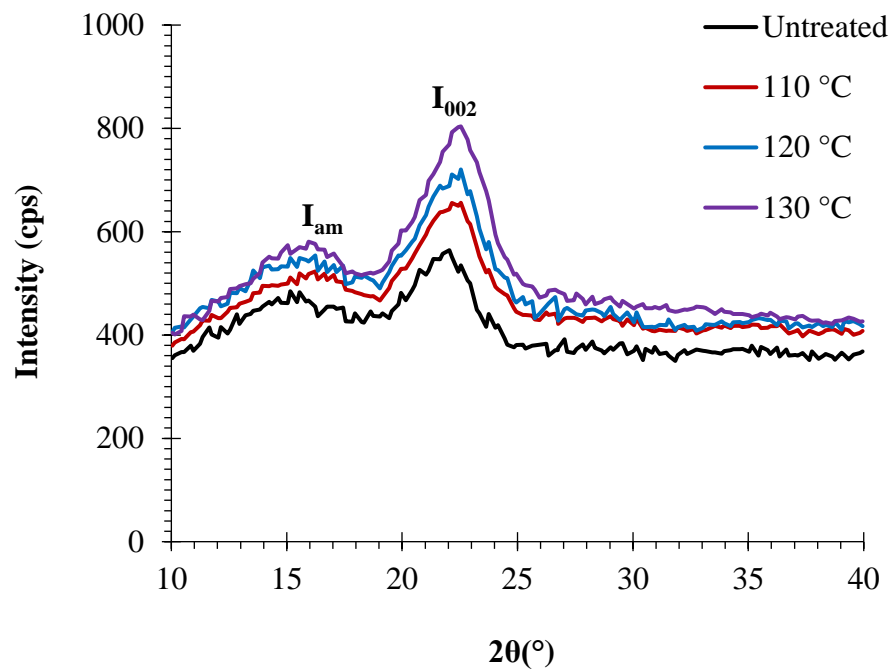

Supplement: Supplemental Information 6 [file peerj-09-12026-s006.pdf]
